# A powerful statistical method identifies novel loci associated with diastolic blood pressure triggered by nonlinear gene-environment interaction

**DOI:** 10.1186/1753-6561-8-S1-S61

**Published:** 2014-06-17

**Authors:** Honglang Wang, Tao He, Cen Wu, Ping-Shou Zhong, Yuehua Cui

**Affiliations:** 1 Department of Statistics & Probability, Michigan State University, 619 Red Cedar Rd. Rm C432, East Lansing, MI 48824, USA

## Abstract

The genetic basis of blood pressure often involves multiple genetic factors and their interactions with environmental factors. Gene-environment interaction is assumed to play an important role in determining individual blood pressure variability. Older people are more prone to high blood pressure than younger ones and the risk may not display a linear trend over the life span. However, which gene shows sensitivity to aging in its effect on blood pressure is not clear. In this work, we allowed the genetic effect to vary over time and propose a varying-coefficient model to identify potential genetic players that show nonlinear response across different age stages. We detected 2 novel loci, gene *MIR1263 *(a microRNA coding gene) on chromosome 3 and gene *UNC13B *on chromosome 9, that are nonlinearly associated with diastolic blood pressure. Further experimental validation is needed to confirm this finding.

## Background

The genetic basis of a complex trait often involves multiple genetic factors functioning in a coordinated manner. The extent to which our genetic blueprint expresses also depends on the interactions between genetic and environmental factors. Increasing evidence shows the importance of gene-environment (G × E) interactions in determining the risk of a variety of diseases such as respiratory diseases [1], obesity [2], and psychiatric disorders [3]. For a review of G × E interaction, see the work of Hunter [4]. The empirical evidence underscores the importance of developing novel statistical approaches to identify major genetic players that are sensitive to environmental stimuli and to further understand how they function.

Blood pressure is a heritable trait influenced by several biological pathways sensitive to environmental stimuli. High blood pressure, or hypertension, affects more than 1 billion people worldwide. It damages an individual's body in many ways over time, leading to heart disease, stroke, kidney failure, and other health problems [5]. Age is known to be a risk factor for high blood pressure. Systolic blood pressure rises with age, whereas the diastolic blood pressure tends to fall. For people with preexisting high blood pressure, this age-related pattern occurs even if the blood pressure is well controlled with medication [6]. The reasons why blood pressure changes with age are still poorly understood, but are a topic of intense research. Thus, age should be an important predictor when searching for genetic players responsible for hypertension. However, few studies have considered an age-dependent mechanism in their analysis.

The genetic response to age in blood pressure fits in well with the classical G × E interaction framework. G × E interaction typically refers to the manner in which genotypes influence phenotypes differently in different environments [7]. From a biological point of view, G × E interaction can be better viewed as the genetic responses to environment changes or stresses [8]. Statistically, interaction is considered as a departure from additivity when fitting a linear regression model with 1 or more product terms, for example,

(1) $$Y=\alpha_{0}+\alpha_{1}X+\beta_{1}G+\beta_{2}XG+\varepsilon =\left(\alpha_{0}+\alpha_{1}X\right)+\left(\beta_{1}+\beta_{2}X\right)G+\epsilon$$

where *Y *is a quantitative trait (diastolic blood pressure in this analysis), *G *is the genetic variable, *X *is the environmental variable (age), and is the error term. This is a classical linear model for G × E interaction analysis. As can be seen, equation (1) automatically assumes a linear interaction mechanism between *G *and *X *because the coefficient for *G *is a linear function in *X*. However, the contribution of the same gene to blood pressure may be quite different at different age levels. This nonlinear penetrance can be well understood by a statistical varying-coefficient (VC) model [9]. VC models allow the coefficients to change smoothly and nonlinearly with other variables so that one can explore the dynamic feature of a response over time with great flexibility and nice interpretability [10].

In this work, we applied VC models to detect genetic variants associated with diastolic blood pressure from the Genetic Analysis Workshop 18 (GAW18) data with 142 unrelated individuals. We allowed the contribution of genetic variants to blood pressure to vary over time via varying coefficients. We further proposed a sequence of hypothesis tests to evaluate whether the effect of a genetic variant is sensitive to aging, and if it is, is it in a linear or nonlinear fashion? Using this analysis, we identified 2 novel loci that show nonlinear effects over time to affect blood pressure.

## Methods

### The model

The nonlinear VC model is defined as

(2) $$Y=m\left(X,G\right)+\sigma \left(X\right)\epsilon$$

for given (X,G) and the response *Y *with E(ϵ|X,G)=0 and $Var\left(\epsilon \text{|}X,G\right)=1;$$\sigma^{2}\left(X\right)=Var\left(Y|X,G\right)$ is the conditional variance function. The mean function is defined as m(X,G)=α(X)+β(X)G, where $\beta \left(X\right)$ is a smoothing function in *X*. Under the VC modeling framework, the effect of a gene is allowed to vary as a function of environmental factors, either linearly or nonlinearly, captured by the model itself. Thus, the VC model has the potential to dissect the nonlinear penetrance of genetic variants. Here we also allow nonlinear function of *X *with *Y *modeled by $\alpha \left(X\right)$. This nonlinear term adjusts the nonlinear effect of *X *when estimating the nonlinear effect of β(X). If we take $\alpha \left(X\right)=\alpha_{0}+\alpha_{1}X,$ equation (1) is just a special case of the VC model when $\beta \left(X\right)=\beta_{1}+\beta_{2}X$.

### Hypothesis testing

The following list shows all 4 mean models involved in our analysis.

• Model 1: m(X,G)=α(X), no genetic effect at all;

• Model 2: m(X,G)=α(X)+βG, linear genetic effect without interaction;

• Model 3: $m\left(X,G\right)=\alpha \left(X\right)+\left(\beta_{0}+\beta_{1}X\right)G,$ linear genetic effect with interaction; and

• Model 4: m(X,G)=α(X)+β(X)G, nonlinear genetic effect.

We first assess whether the genetic coefficients vary with × by formulating the following hypotheses,

$$H_{0}^{1}:\beta \left(X\right)=\beta \;\text{for}\;\text{some}\;\text{constant}\;\beta \;\text{vs}.\;H_{a}^{1}:\beta \left(\text{X}\right)\neq \beta \;\text{for}\;\text{any}\;\beta$$

Rejecting the null indicates that potential gene-age (G × age) interaction may exist. Otherwise, we conclude there is no G × age effect and we fit mean model 2 to test for association. Because the traditional linear interaction model given in equation (1) is a special case of the proposed VC model, we next test significance of a linear effect if the above null is rejected, by formulating the following hypotheses,

$$\{\begin{array}{c} H_{0}^{2}:\beta \left(X\right)=\beta_{0}+\beta_{1}X\;\text{for}\;\text{some}\;\text{constants}\;\beta_{0},\beta_{1}\\ H_{\text{a}}^{2}:\beta \left(\text{X}\right)\neq \beta_{0}+\beta_{1}\text{X}\;\text{for}\;\text{any}\;\beta_{0},\beta_{1} \end{array}$$

Failure to reject the null indicates that there is a linear G × age effect, so we fit mean model 3 to assess association. Otherwise, we conclude that the G × age interaction is nonlinear. We then assess the nonlinear genetic effect over age by formulating the hypotheses,

$$H_{0}^{3}:\beta \left(X\right)=0,\text{vs}\;H_{a}^{3}:\beta \left(X\right)\neq 0$$

The rejection of the null indicates that the genetic effect is sensitive to age in a nonlinear fashion. The sequence of hypothesis tests stated above was suggested by Ma et al [9] for optimal power to detect association.

### Model implementation

We fit the varying coefficients with a B-spline technique for both α(⋅) and β(⋅) functions. The *X *variable was first transformed to make it more evenly distributed on each subinterval used in the B-spline smoothing technique. The great advantages of B-spline estimation over other nonparametric techniques are simple implementation and fast computing [9]. For each single-nucleotide polymorphism (SNP), α(X) in mean model 1 was estimated by considering the following least square problem,

$$argmin_{{\left\{\lambda_{s}\right\}}_{s=1}^{N+p+1}}\sum_{i=1}^{n}{\left\{Y_{i}-\sum_{s=1}^{N+p+1}\lambda_{s}B_{s}\left(X_{i}\right)\right\}}^{2}$$

The estimated α(X) has the form $\hat{\alpha }\left(x\right)=\sum_{s=1}^{N+p+1}\lambda_{s}B_{s}\left(x\right),$ where *N *is the number of interior knots, *p *is the degree of B-splines, and $G_{p}={\left\{B_{s}\right\}}_{s=1,2,\ldots ,N+p+1}$ is the set of basis B-splines with degree *p*. For selecting the number of knots *N *and the degree *p *of the B-splines, we used the Bayesian information criterion (BIC),

$$argmin_{N,p}BIC\left(N,p\right)=argmin_{N,p}log\left({\hat{\tau }}^{2}\right)+\left(N+p\right)log\left(n\right)/n,$$

where τ^2=1/n∑i=1nYi-m^Xi,Gi2 Then the same number of knots $N_{\alpha }$ and degree $p_{\alpha }$ were applied to estimate function α(X) when fitting mean models 2 to 4.

For mean model 4, the coefficient functions α(x) and β(x) were estimated by,

$$argmin_{{\left\{\theta_{t}\right\}}_{t=1}^{N_{\alpha }+p_{\alpha }+1},{\left\{\lambda_{s}\right\}}_{s=1}^{N+p+1}}\overset{n}{\underset{i=1}{\sum }}{\left\{Y_{i}-\overset{N_{\alpha }+p_{\alpha }+1}{\underset{t=1}{\sum }}\theta_{t}B_{t}\left(X_{i}\right)-\overset{N+p+1}{\underset{s=1}{\sum }}\lambda_{s}B_{s}\left(X_{i}\right)G_{i}\right\}}^{2}$$

Thus we have $\hat{\alpha }\left(x\right)=\sum_{t\;=\;1}^{N_{\alpha }+p_{\alpha }+1}{\hat{\theta }}_{t}B_{t}\left(x\right)$ and $\hat{\beta }\left(x\right)=\sum_{s=1}^{N_{\beta }+p_{\beta }+1}{\hat{\lambda }}_{s}B_{s}\left(x\right),$ where $N_{\beta }$ and $p_{\beta }$ are also selected following the above BIC criterion.

The error term σ(X) can be assumed homogeneous following a normal distribution or heterogeneous without assuming a parametric distribution. When the homogeneous assumption is made, the likelihood ratio test can be applied to assess the significance of $H_{0}^{3}$. Otherwise $\sigma^{2}\left(x\right)$ can be nonparametrically estimated using the spline approximation $\sigma^{2}\left(x\right)\approx \sum_{s=1}^{N+p+1}v_{s}B_{s}\left(x\right)$ and defining σ^2(x)= ∑s=1N+p+1v^sBs(x) as the spline estimate, where v^s=v^1,v^2,⋯,v^N+p+1T minimizes ∑i=1nϵ^2Xi-∑s=1N+p+1vsBsXi2; that is, v^=argminvϵ^2-BvT(ϵ^2-Bv), where ϵ^2=Y1-m^X1,G12,⋯,Yn-m^Xn,Gn2T and

$$B=\left(\begin{array}{c} {\text{B}}_{1}\left({\text{X}}_{1}\right)\\ {\text{B}}_{1}\left({\text{X}}_{2}\right)\\ \cdots \\ {\text{B}}_{1}\left({\text{X}}_{\text{n}}\right) \end{array}\begin{array}{c} {\text{B}}_{2}\left({\text{X}}_{1}\right)\\ {\text{B}}_{2}\left({\text{X}}_{2}\right)\\ \cdots \\ {\text{B}}_{2}\left({\text{X}}_{\text{n}}\right) \end{array}\begin{array}{c} \cdots \\ \cdots \\ \cdots \\ \cdots \end{array}\begin{array}{c} {\text{B}}_{\text{N}+\text{p}+1}\left({\text{X}}_{1}\right)\\ {\text{B}}_{\text{N}+\text{p}+1}\left({\text{X}}_{2}\right)\\ \cdots \\ {\text{B}}_{\text{N}+\text{p}+1}\left({\text{X}}_{\text{n}}\right) \end{array}\right).$$

Thus we have v^=BTB-1BTϵ^2, and σ^2X1,⋯,σ^2XnT=Bv^=BBTB-1BTϵ^2. Wild bootstrap can be applied to assess the significance of $H_{0}^{3}$[11].

## Results

We applied the above models to the GAW18 genome-wide association data. We focused our analysis on diastolic blood pressure (DBP) to identify any genetic players that can explain the variability of DBP triggered by nonlinear genetic penetrance over time. We treated DBP as the response *Y *and age as the *X *variable. The genetic variable *G *is coded following an additive model, that is, *G *= 1, 0, −1, corresponding to genotype *AA, Aa, aa*, respectively. In total, 142 individuals and 388,099 SNPs were left after removing SNPs with a minor allele frequency less than 0.05. These SNPs are distributed on odd-numbered chromosomes from chromosome 1 to chromosome 21.

**Figure 1:**
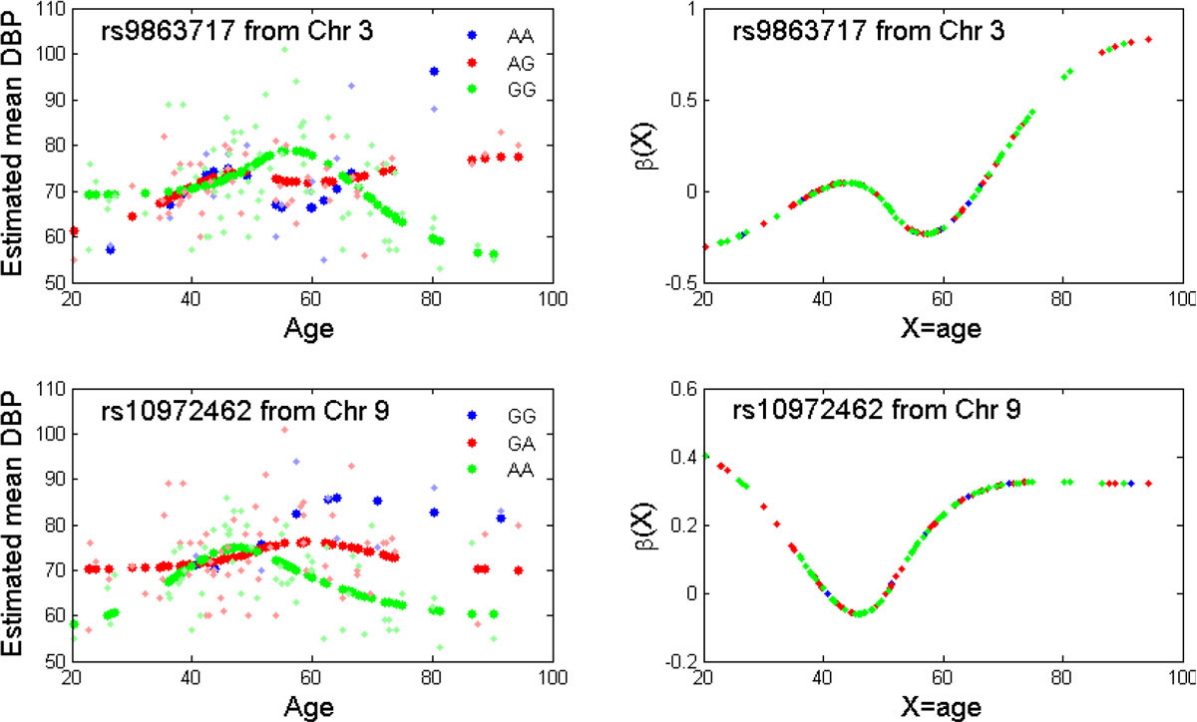
**Manhattan plots of the *p *values for assessing significance of:**.A, $H_{0}:\beta =0$ by fitting mean model 2; (B) $H_{0}:\beta_{0}=\beta_{1}=0$ by fitting mean model 3; and (C) $H_{0}:\beta \left(X\right)=0$ by fitting mean model 4. Solid red, blue, and gray lines correspond to significance levels of $10^{-7}$, $10^{-6}$, and $10^{-5}$, respectively.

Figure 1 shows the Manhattan plots of *p *values when assessing significance by fitting different models. The overall *p *value patterns for the 3 models are quite similar. Two known and 1 unknown gene show strong nonlinear genetic effects (indicated by small *p *values in columns 7 and 8 in Table 1). A strong signal was detected in chromosome 3 containing a microRNA coding gene, *MIR1263*, and in chromosome 9 containing the gene *UNC13B. MIR1263 *may play a regulatory role. The signals at gene *UNC13B *are quite consistent for the 3 models. This gene was reported to be related with increased risk of nephropathy in patients with type 1 diabetes. Nephropathy accounts for 40% of end-stage renal disease and is associated with high cardiovascular morbidity and mortality [12].

**Table 1:** List of SNPs with *p *value <5 × 10^−^^7^

rs ID	Gene name	Chr	$p_{41}$	$p_{31}$	$p_{21}$	$p_{42}$	$p_{43}$
rs1086097	*MIR1263*	3	1.9 × 10^−7^	0.009	0.90	4.97 × 10^−8^	1.08 × 10^−6^
rs686697	*MIR1263*	3	3.4 × 10^−7^	0.005	0.76	9.24 × 10^−8^	3.41 × 10^−6^
rs483558	unknown	3	4.7 × 10^−7^	0.007	0.76	1.30 × 10^−7^	3.72 × 10^−6^
rs9863717*	unknown	3	4.96 × 10^−8^	0.009	0.95	1.23 × 10^−8^	2.55 × 10^−7^
rs1575160*	unknown	3	8.7 × 10^−8^	0.011	0.70	2.33 × 10^−8^	3.76 × 10^−7^
rs723877	*UNC13B*	9	4.3 × 10^−7^	1.25 × 10^−6^	2.37 × 10^−5^	6.1 × 10^−4^	0.02
rs10972462*	*UNC13B*	9	9.5 × 10^−8^	6.96 × 10^−7^	1.31 × 10^−5^	2.3 × 10^−4^	0.007

*Indicates significant SNPs after Bonferroni correction. $p_{41}$, $p_{31},$ and $p_{21}$ are *p *values for testing $H_{0}^{3}:\beta \left(X\right)=0$ (mean model 4 vs. mean model 1), $H_{0}^{2}:\beta_{0}=\beta_{1}=0$ (mean model 3 vs. mean model 1), and $H_{0}^{1}:\beta =0$ (mean model 2 vs. mean model 1), respectively; $p_{42}$ and $p_{43}$ are *p *values for testing $H_{0}:\beta \left(X\right)=\beta_{0}$ and $H_{0}:\beta \left(X\right)=\beta_{0}+\beta_{1}X,$ respectively. Small values of $p_{42}$ and $p_{43}$ indicate nonlinear G × E effect. The small *p *values for those 3 SNPs with unknown gene names in chromosome 3 are in high linkage disequilibrium with those in gene *MIR1263*.

**Figure 2:**
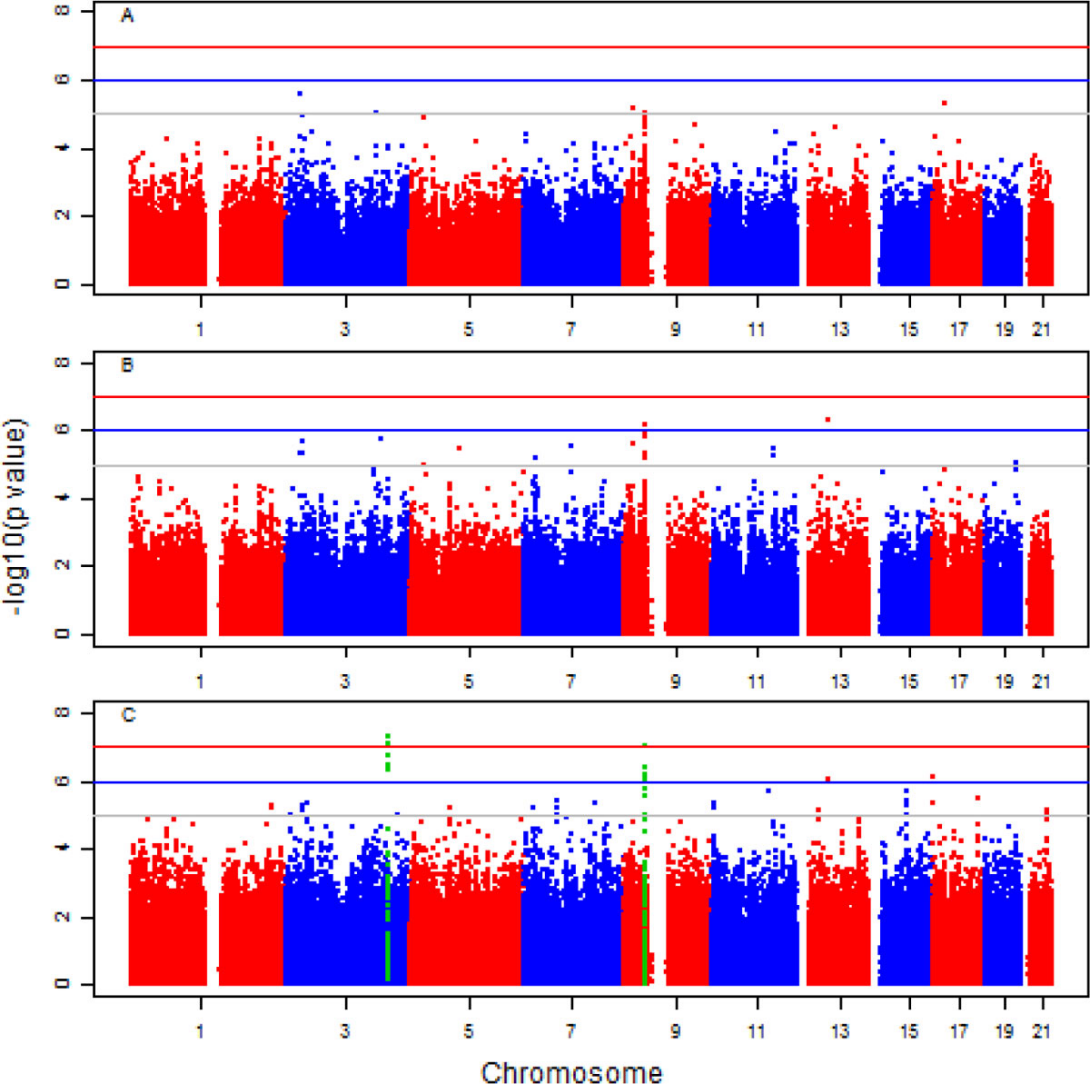
**Fitted mean function and the estimated varying coefficients**. Shown are effects for SNP rs9863717 in gene *MIR1263 *(chr3) and SNP rs10972462 in gene *UNC13B *(chr9). The observed data are shown in lighter color in the background.

Table 1 lists SNPs with *p *values less than 5 × 10^−7^. These SNPs show strong nonlinear effects over time to affect DBP, especially for SNPs in chromosome 3 (indicated by small $p_{42}$ and $p_{43}$ in Table 1). These SNPs can be easily missed by fitting traditional linear models. For illustration purposes, Figure 2 shows the fitted mean DBP function and the genetic effects of 2 SNPs in genes *MIR1263 *and *UNC13B*. For SNP rs9863717 in gene *MIR1263*, DBP decreases after age 55 years for individuals carrying genotype GG, whereas it increases for individuals carrying genotype AA. Thus, for a senior person who carries genotype AA at this locus, the chance to develop hypertension is higher than for others. For SNP rs10972462 in gene *UNC13B*, large DBP variability among the 3 genotype groups is observed after age 50 years, and a decreasing pattern is observed roughly after age 65 years. Among the 3 genotype groups, DBP is higher in the GG group, followed by the GA and AA groups. From the prevention and therapeutic point of view, people carrying genotype GG at rs10972462 locus should pay special attention after age 50 years, and so should those carrying AA genotype at rs9863717 locus after age 65 years.

## Discussion and conclusions

In this work, we proposed to model the genetic effect as a nonlinear function of age. It is clear that the classical linear model, with or without interaction, is just a special case of the VC model. However, the VC model has the flexibility to capture potential nonlinear genetic effects over time. Evidence of nonlinear genetic effects has been reported previously. For example, Laitala et al [13] reported the curvilinear genetic effect on interindividual differences in coffee consumption over age. In a study of congenital scoliosis in mice [14], the authors found that mutations in genes *HES7 *and *MESP2 *are sensitive to different degrees of hypoxia, which is responsible for a nonlinear increase in the severity and penetrance of vertebral defects. Our analysis identified 2 novel loci associated with DBP with nonlinear genetic effects. They can be missed by the traditional linear interaction model. However, because statistical significance does not necessarily imply causality, further experimental validation is needed to confirm the finding.

As shown in Ma et al [9], the VC model loses power because of high degrees of freedom in the test in cases where the genetic effect is not very complex, such as in a linear form. Thus one should assess constant or linear effects first, followed by fitting the corresponding model suggested by the results of the tests. In this analysis, we found that the coefficients are constant for most SNPs.

Note that the function α(X) models the overall mean of DBP over time when there is no genetic effect. When a linear structure for $\alpha \left(X\right)\left(=\alpha_{0}+\alpha_{1}X\right)$ is forced, we observe inflated signals for testing $H_{0}:\beta \left(X\right)=0$. Thus, the incorporation of this nonlinear function can largely reduce false positives. In this analysis we coded the genetic variable G in an additive fashion, although other disease models such as dominant or recessive can also be assumed, while the optimal one can be selected based on a model selection criterion such as BIC.

## Competing interests

The authors declare that they have no competing interests.

## Authors' contributions

HLW conducted the analysis and drafted the manuscript; TH, CW, and PSZ participated the discussion and helped with the analysis; YHC conceived the idea and wrote the manuscript. All authors read and approved the final manuscript.
